# Low-loss microwave photonics links using hollow core fibres

**DOI:** 10.1038/s41377-022-00908-3

**Published:** 2022-07-07

**Authors:** Xi Zhang, Zitong Feng, David Marpaung, Eric Numkam Fokoua, Hesham Sakr, John Richard Hayes, Francesco Poletti, David John Richardson, Radan Slavík

**Affiliations:** 1 Optoelectronics Research Centre, University of Southampton, Southampton, SO17 1BJ UK; 2 Laser Physics and Nonlinear Optics Group, Faculty of Science and Technology, MESA + Institute for Nanotechnology, University of Twente, Enschede, 7500 AE The Netherlands

**Keywords:** Fibre optics and optical communications, Microwave photonics

## Abstract

There are a host of applications in communications, sensing, and science, in which analogue signal transmission is preferred over today’s dominant digital transmission. In some of these applications, the advantage is in lower cost, while in others, it lies in superior performance. However, especially for longer analogue photonics links (up to 10 s of km), the performance is strongly limited by the impairments arising from using standard single-mode fibres (SSMF). Firstly, the three key metrics of analogue links (loss, noise figure, and dynamic range) tend to improve with received power, but this is limited by stimulated Brillouin scattering in SSMF. Further degradation is due to the chromatic dispersion of SSMF, which induces radio-frequency (RF) signal fading, increases even-order distortions, and causes phase-to-intensity-noise conversion. Further distortions still, are caused by the Kerr nonlinearity of SSMF. We propose to address all of these shortcomings by replacing SSMFs with hollow-core optical fibres, which have simultaneously six times lower chromatic dispersion and several orders of magnitude lower nonlinearity (Brillouin, Kerr). We demonstrate the advantages in this application using a 7.7 km long hollow-core fibre sample, significantly surpassing the performance of an SSMF link in virtually every metric, including 15 dB higher link gain and 6 dB lower noise figure.

## Abstract (web summary)

We demonstrate analogue photonics links using hollow-core fibres, showing they significantly surpass the performance of standard fibre analogue links in every metrics of link gain, noise figure, and dynamic range.

## Graphical abstract

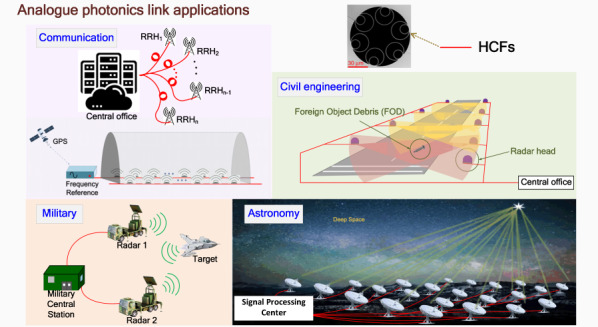

## Introduction

**Fig. 1:**
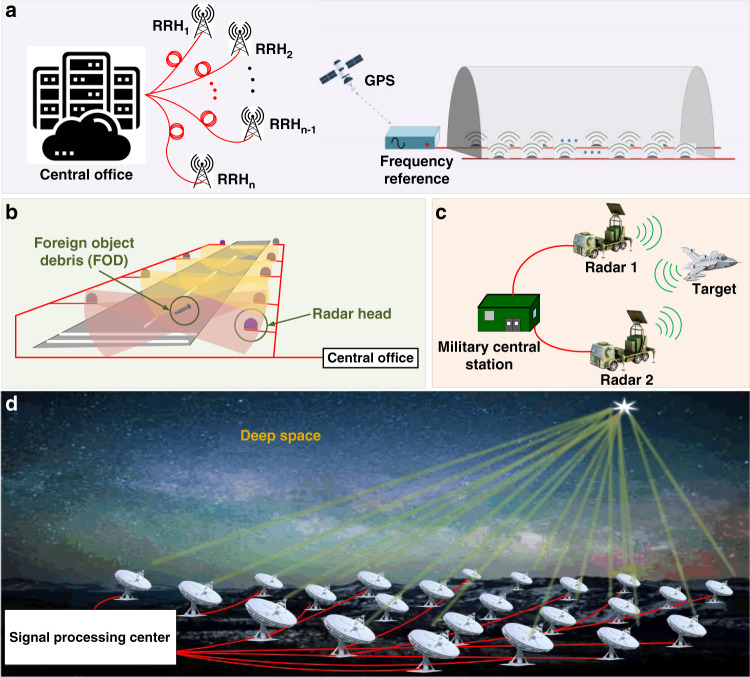
Examples of applications using long-haul (>1 km) APLs. Fibre links are shown as red lines. **a**, **b** Examples of APL use in civil applications, and **c**, **d** in military and science applications, respectively. **a** Connection of a large number of remote radio heads (RRH) for wireless communications providing connectivity, but also timing/positioning (e.g., in a tunnel). **b** Monitoring and identification of foreign objects over a large area such as a runway. **c** Military (high dynamic range) radar systems. **d** Radio antenna arrays for exploring objects in deep space

Data transmission over optical fibres has enabled the Internet as we know it today. Optical fibres are used for connections spanning from several metres (e.g., between server racks in a data centres) to 10,000 km in intercontinental links. Data transmitted through these fibres are almost exclusively encoded digitally, requiring imprinting of the digital signal on an optical wave that is transmitted through the fibre, with the signal subsequently photodetected and digitized at the receiver. The key advantage of digital systems is their tolerance to system impairments, achieved thanks to the digital signal processing, e.g., using ubiquitous forward-error correction algorithms. There are, however, many applications in which analogue signal transmission may be preferred. In communications, analogue photonics links (APLs) enable use of a simple and low-power-consumption Remote Radio Head (RRH) connected via fibre to the central office (Fig. 1a), as there is no digitization or digital signal processing required at the RRH. In scenarios like 5G and beyond, where a large number of RRHs is needed (e.g., hundreds to thousands), this brings significant advantages in terms of cost and network management. For example, a cost saving by a factor of four was predicted^1^ in a Centralized Radio Access Network (C-RAN) when using an APL as opposed to digitally-encoded transmission. Another example where a large number of RRHs are needed is in providing internet connectivity and positioning in high-speed railways, along motorways, and in tunnels, see Fig. 1a. Further examples of APL^2–13^ use range from civil, military, to science applications. They include examples such as foreign object debris (FOD) detection on airport runways (Fig. 1b), improving safety and reducing flight delays significantly. To achieve the required object spatial resolution of 0.01 m^2^ (ref. ^6^), millimetre waves at 80–110 GHz frequencies are typically used^7^. As compared to digital systems, APLs are more able to handle these high frequencies, as they, for example, do not require high-frequency digitizers that are costly and add digital quantization noise. Employing APLs for antenna remoting is also attractive in military and warfare applications (Fig. 1c), where digital transmission cannot meet the very stringent technical requirements, while APL in principle can. For example, anti-jamming radar requires a spurious-free dynamic range (SFDR) as high as 120–130 dB·Hz^2/3^ ^8^, which in principle can be achieved by a well-optimized APL, but which is almost impossible to achieve with digital transmission as such a system would put unrealistic requirements on the digitizing circuits. An example of APL use in science is shown in Fig. 1d. In deep space radio astronomy, APLs can deliver signals to remote antennas and distribute local oscillator signals, potentially enabling improved resolution and sensitivity in facilities such as the Allen Telescope Array (ATA)^10^, Atacama Millimetre Array (ALMA)^11^, the Low-Frequency Array (LOFAR)^12^ and the Square Kilometre Array (SKA)^13^.

In most of the above-mentioned applications, the fibre length can reach several kilometres or even tens of kilometres. At these distances, APLs have to cope with the various nonlinear and linear impairments present in today’s standard single-mode fibres (SSMFs). There is also often a trade-off between these impairments, e.g., a reduction of the chromatic dispersion increases nonlinear interactions. Among the various nonlinear impairments, it is primarily stimulated Brillouin scattering (SBS), which limits the maximum power that can be launched or delivered via the SSMF, limiting the APL performance in terms of its two key characteristics: loss and signal-to-noise ratio^14^. Besides the loss, SBS also adds noise over a broad frequency range, although the most significant degradation occurs around 10 GHz, which is the acoustic phonon frequency in SSMFs^15–17^. Another nonlinear fibre impairment is the Kerr nonlinearity^15,18–20^, which produces self-phase modulation (SPM), cross-phase modulation (XPM) and four-wave mixing (FWM). SPM affects single-channel systems, causing intermodulation distortions and adding noise. In wavelength-division-multiplexed (WDM) systems, further distortions are observed due to nonlinear interaction between the individual channels through XPM and FWM^19,20^. In terms of linear impairments, it is mainly the chromatic dispersion, which may cause signal fading^21^, especially in long (>km) APLs or when high frequencies such as microwave and millimetre-wave are used. For example, the first fading dip frequency for an amplitude-modulated signal propagating through 1 km SSMF is around 60 GHz, while for a 50 km fibre it is only 9 GHz. In addition, second-order harmonic and intermodulation distortions are enhanced by chromatic dispersion in multi-octave systems^22^.

SBS in long-haul APLs can be reduced via engineering of the transmission fibre, e.g., concatenating fibres with different SBS frequencies^23,24^, the use of few mode fibres^25^, via aluminium doping^26^, or use of optical isolators along the fibre length^23,27^. Other strategies involve engineering of the transmitter, e.g., using frequency comb sources^28,29^, dithering^30^, or optimized modulation formats^31^. These techniques have shown increased power handling, e.g., by up to 8.8 dB for a 20-km long fibre^23^, however, further scaling of the power seems challenging. Another mitigation method is to optically amplify the signal prior to photodetection. Although this reduces the insertion loss, it leads to degradation of the APL noise figure, as noise from the optical amplifier is directly photodetected^32^. Linear fibre impairments can also be addressed, e.g., single-sideband modulation avoids chromatic dispersion-induced fading^33^. However, this requires more advanced transmitters e.g. based upon dual-drive Mach-Zehnder modulators (DD-MZMs)^34^ or IQ MZMs^33^ in which the input signal is first split and subsequently delivered to multiple electrodes, increasing complexity. Losing the power of the other sideband is also detrimental to the gain, noise figure, and SFDR of the entire link and the loss in performance cannot easily be restored just by amplification. All the discussed compensation methods are not as universal or as cost-effective as using a single optical fibre or a relatively simple transmitter/receiver, as they require post-fabrication and post-packaging adaptation and different designs may be needed for different fibre lengths or data formats. An ideal fibre would not require any of these techniques and could be used directly with arbitrary length and be incorporated in robust protective cabling—similar to what is available today for SSMF.

In this work, we suggest replacing the SSMF in APLs with a new type of optical fibre that does not guide light through silica glass, but through a central hollow core region. These fibres are referred to as hollow-core optical fibres (HCFs). Recently, the attenuation of HCFs has approached that of SSMF (currently 0.22 dB/km^35^ in HCF versus 0.14 dB/km in SSMF^36^) and is predicted to be further reduced to levels below that of the best SSMF^37^. As light does not propagate in HCF through the glass, nonlinearity is strongly suppressed by 2–3 orders of magnitude in air-filled HCFs^38^, which is achieved simultaneously with low chromatic dispersion, e.g., below 3 ps/nm/km^39^. This avoids the above-mentioned dispersion/nonlinearity trade-off present in SSMF. As an additional benefit, light propagates through an HCF at almost the speed of light in vacuum, reducing fibre-imposed latency by ~30% as compared to SSMF. Due to these unique and enabling properties, significant effort has been put into their fabrication and cabling and they have recently become commercially available (e.g., by Lumenisity Ltd, UK (https://lumenisity.com/)). Relevant HCF and SSMF properties are summarized in Table 1. The HCF loss, nonlinearity, and chromatic dispersion we have referred to above were achieved in the nested antiresonant nodeless fibre (NANF) HCF geometry^40^. Although these HCFs are intrinsically multi-moded, they can be designed so that all higher-order modes suffer prohibitively high losses, making them effectively single-moded. For example, a recent result^35^ with a 5-tube NANF type HCF promises higher-order mode losses above 2000 dB/km, making these fibres robustly single-moded over lengths in excess of several tens of metres.

To demonstrate the advantages of HCFs in APLs, we performed a set of comprehensive measurements using the well-known intensity-modulation direct-detection (IM-DD) APL. We characterize the back-to-back performance and then use a 7-km-long NANF type HCF and SSMF. In particular, we study how the SSMF and HCF influence the three key APL parameters of the link gain, noise figure, and dynamic range. Notably, when replacing the SSMF with our HCF we achieved 15 dB increase in the link gain, >6 dB noise figure reduction, 35 dB lower FWM products, and 2-dB reduced chromatic dispersion-induced fading.

## Results

### Link gain

**Fig. 2:**
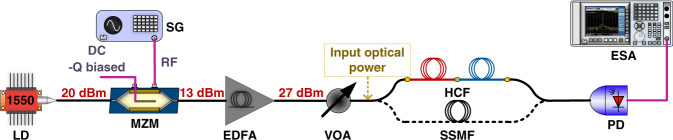
Quadrature-biased APL set-up. LD, laser diode; EDFA, erbium-doped fibre amplifier; MZM, Mach-Zehnder amplitude modulator; SG, signal generator; VOA, variable optical attenuator; PD, photodiode; ESA, electrical spectrum analyzer. Details of all components are given in the ‘Methods’ section

First, we built an IM-DD APL with a 20-GHz bandwidth Mach-Zehnder modulator and a 20-GHz bandwidth photodetector capable of receiving up to 18 dBm of optical power. To boost the power, we used a low-noise erbium-doped fibre amplifier (EDFA). Set-up details are described in the ‘Methods’ section. We operated the transmitter either at the quadrature point (Q-biased), Fig. 2, or using low-biasing (L-biased). At Q-biasing, the modulator shows the best linearity. L-biasing reduces the carrier and thus can avoid detector saturation, enabling more RF power to be received, improving the link gain and thus lowering the noise figure^41^. L-biasing, however, generates odd-order distortions, so can only be used when these can be tolerated.

The fibres employed here were a 7.7 km length of HCF and, for comparison, also an SSMF of a similar length (7.4 km). We measured the SBS threshold of our SSMF to be 12.5 dBm, which represents the maximum CW optical power the fibre can transmit. As this is significantly below what today’s lasers and detectors can generate and detect, respectively, the power-handling capability of the SSMF represents a bottleneck in the achievable APL performance. The ‘Methods’ section gives further fibre samples details.

The most effective way to simultaneously achieve low link loss, low noise figure, and high spurious-free dynamic range is to receive as high optical power as possible at the photodiode (PD)^14,42^. Thus, we targeted to get as close as possible to the maximum operating power of our PD of 18 dBm. To enable this, we fixed the transmitter output power at 27 dBm and controlled the power launched into the link via a variable optical attenuator (VOA).

#### Q-biased APL

**Fig. 3:**
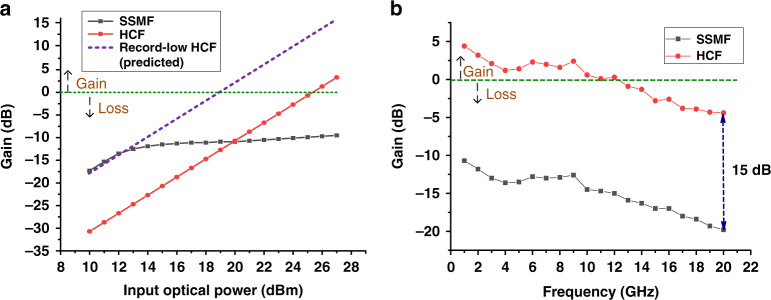
Measured Q-biased APL gain (set-up shown in Fig. 2). **a** Gain is plotted as a function of the optical power launched into the transmission fibre. The input signal has a frequency of 1 GHz. The measured gain with the SSMF transmission fibre is shown as a solid line with square symbols) and the measured gain with the HCF transmission fibre is shown as a solid line with circle symbols. The predicted gain considering a HCF transmission fibre with the lowest-published attenuation to date is shown as a dashed line. Dashed black arrows indicate net gain or loss. **b** Measured gain over the frequency range of 0–20 GHz obtained for the maximum received power as limited by SBS (SSMF APL) and photodiode (HCF APL). The HCF APL shows 15-dB higher gain than the SSMF APL

Figure 3 shows the experimental results. With SSMF, we see different behaviour below and above the SBS threshold of 12.5 dBm. Below this threshold, the APL RF gain increases by 2 dB for every 1-dB increase in the launched optical power, expected from a corresponding 1-dB optical power increase at the PD. However, above the SBS threshold, the link gain increases at a significantly lower rate, as the power delivered to the PD is essentially constant (any increase in the optical power sent through the SSMF is scattered backwards due to the SBS and thus does not reach the PD). The maximum gain reaches −9.5 dB at 1 GHz. With HCF, the link gain increases linearly as expected (by 2 dB for every 1-dB increase of input optical power) until we reach the maximum power of our PD (18 dBm), showing no SBS limitation. The maximum gain is 4.3 dB at 1 GHz. Figure 3b compares the best performance achieved (limited by SBS in SSMF and our PD power handling for the HCF). We see the HCF-based APL shows more than 15 dB higher link gain over the entire frequency range as compared to the SSMF-based APL, and actually provides a net gain up to 12 GHz with a minimum gain of −5 dB over the entire range. Net gain over a broader frequency range could be achieved, e.g., by using a modulator or a PD with a higher 3-dB bandwidth.

Figure 3a also shows our prediction considering HCF with the state-of-the-art attenuation of 0.22 dB/km^29^ and the state-of-the-art HCF-SSMF pigtail loss (0.15 dB per pigtail^43^) that enables interconnection of HCF with standard off-the-shelf fibre optics components. The total loss of such a pigtailed HCF would be as little as 2.0 dB, which is only 1.1 dB below that of the lowest-loss SSMF (0.14 dB/km). With such a sample, the PD maximum power (18 dBm) would be reached with an incident transmitted power as low as 20 dBm, which is a power level routinely used in today’s fibre optic communications.

Indeed, the gain of the SSMF link could be improved by operating the SSMF below the SBS threshold and using an EDFA in front of the PD. We discuss this configuration later, however, it is worth mentioning that it is often not preferred in APLs, as each receiver would then require an EDFA, which is impractical/costly when considering a large number of RHHs as discussed earlier. As we show later in detail, this solution also suffers from an undesirable higher noise figure.

#### L-biased APL

**Fig. 4:**
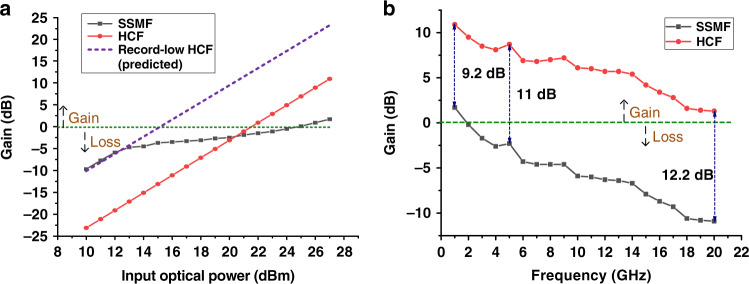
Measured L-biased APL gain. The set-up is identical to that shown in Fig. 2, other than the MZM is low-biased (L-biased). **a** Gain is plotted as a function of the optical power launched into the transmission fibre. The input signal has a frequency of 1 GHz. The measured gain with the SSMF transmission fibre is shown as a solid line with square symbols) and the measured gain with the HCF transmission fibre is shown as a solid line with circle symbols. The predicted gain considering a HCF transmission fibre with the lowest-published attenuation to date is shown as a dashed line. Dashed black arrows indicate net gain or loss. **b** Measured gain over the frequency range of 0–20 GHz obtained for the maximum received power limited by SBS (SSMF APL) and photodiode (HCF APL). The HCF APL shows 9, 11 and 12 dB higher gain than the SSMF APL at frequencies of 1, 5 and 20 GHz, respectively

Figure 4 shows the results we obtained when low-biasing our transmitter (“L-biased” transmitter). Although the gain is improved in both SSMF and HCF APLs, the SSMF APL shows significant degradation due to SBS once the launched power reaches the SBS threshold. Due to this limitation, the HCF link achieves higher link gain, with an advantage of 9 dB or more over the entire frequency range as compared to the SSMF APL. It is worth mentioning that the HCF APL gain is positive over the entire frequency range.

We notice the SSMF APL gain keeps increasing with increased launched power and its slope is actually improving when launching very high powers into the SSMF. We believe that the initial growth is because the modulation side-bands are weaker than the carrier and thus keep growing even when the carrier is above the SBS threshold. At even higher powers, SPM and FWM transfer power from the carrier to the modulation side-bands. For Q-biased APL, the even-order modulation side-bands are suppressed, which we believe is the reason we do not see this as prominently in that case (shown in Fig. 3a).

### Noise figure

**Fig. 5:**
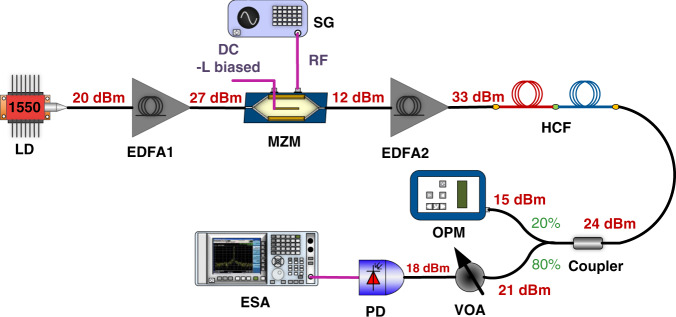
Noise figure optimized HCF APL set-up. The MZM was L-biased and two EDFAs were used to keep the optical power as high as possible (limited by the power handling of the MZM used (500 mW maximum input) along the link, minimizing degradation due to shot noise. LD, laser diode; EDFA, erbium-doped fibre amplifier; MZM, Mach-Zehnder modulator; SG, signal generator; VOA, variable optical attenuator; OPM, optical power metre; PD, photodiode; ESA, electrical spectrum analyzer. Details of the specific components used are given in the ‘Methods’ section

**Fig. 6:**
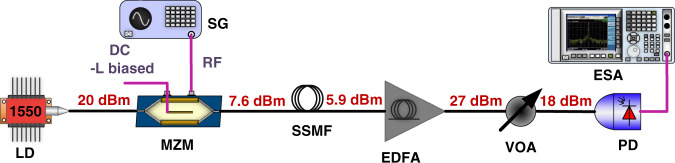
Noise figure optimized (SBS-free) SSMF APL set-up. The MZM was L-biased, the input into the SSMF was kept well below the SBS threshold to avoid signal degradation, and the optical power prior to photodetection was boosted with a low-noise EDFA to raise the signal noise above the thermal noise. LD, laser diode; MZM, Mach-Zehnder modulator; EDFA, erbium-doped fibre amplifier; SG, signal generator; VOA, variable optical attenuator; PD, photodiode; ESA, electrical spectrum analyzer. Details of the specific components used are given in the ‘Methods’ section

Here, we set up both links, HCF and SSMF-based, to achieve the minimum possible noise figure while achieving the same gain. For HCF, we use two EDFAs (before and after the MZM) in the transmitter to minimize the ASE noise (Fig. 5). This configuration allows us to send as much as 33 dBm into the HCF. For the SSMF link, we keep the launch power below the SBS threshold and place an EDFA in front of the PD to obtain the same optical power at the PD and thus also the same link gain (Fig. 6). We use a low-biased transmitter, which generates 7.6 dBm at its output, which is 5 dB below the SBS threshold, ensuring that SBS does not degrade the noise figure. The EDFA was operated at its nominal output power of 27 dBm (ensuring optimum noise performance). Both, the HCF and SSMF link had a VOA in front of the PD to adjust the power to 18 dBm.

**Fig. 7:**
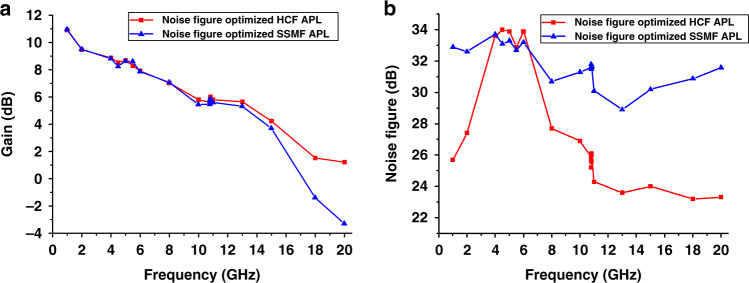
Measured APL performance in terms of gain and noise figure of the noise figure optimized APLs. **a** Gain of noise figure optimized SSMF (triangle symbols) and HCF (square symbols) APLs versus RF input frequency. Gain reduction at higher frequencies is mainly due to the limited bandwidth of the MZM and PD. **b** Corresponding noise figures. At 4–7 GHz range, HCF APL noise figure is limited by the signalling laser RIN due to its relaxation oscillation

The APL link gain and noise figure were measured using the methods described in ref. ^44^ and the ‘Methods’ section and the results are shown in Fig. 7. The gain at frequencies below 15 GHz is almost identical for both links. Above 15 GHz, we start to observe degradation in the SMF APL gain due to dispersion-induced fading, with the first dip expected at 22 GHz (Table 1). As shown in Table 1, the first dispersion-induced fading dip in the HCF APL should occur at a significantly higher frequency (65 GHz), allowing the link to operate with minimal distortion up to about 60 GHz. Above this frequency, however, techniques developed for SSMF APLs such as single-sideband modulation^33^ would need to be adopted even for the HCF APL.

**Table 1:** Key properties of SSMF and HCF (NANF type) relevant for APLs

		SSMF	HCF
	
Fibre loss (lowest published value)		0.14 dB/km^36^	0.22 dB/km^35^
SBS linear gain		3 × 10^−11^ (in silica)^40^	4 × 10^−14^ (in air)^40^
Chromatic dispersion @ 1550 nm^a^		17 ps/(nm·km)	2 ps/(nm·km)^39^
The first fading dip frequency^21^^,a^	1 km	61 GHz	177 GHz
	7.5 km	22 GHz	65 GHz
	50 km	9 GHz	25 GHz
Nonlinear coefficient^a^		1.3 (W km)^−1^ ^51^	5 × 10^−4^ (W km)^−1^ ^51^

^a^Chromatic dispersion in SSMF can be reduced via the design, increasing the first fading dip frequency. However, this causes undesired increase in the nonlinear coefficient

The noise properties of both APLs are shown in Fig. 7b. Around 5 GHz, both APLs seem to be limited by the relaxation oscillation RIN noise peak of the transmitter laser that occurs in this frequency range. Subsequently, EDFA noise does not play a dominant role in this frequency range. However, away from this peak (which can be reduced if a lower-RIN signalling laser is used, e.g. a 1782 DWDM laser from Emcore has RIN below −163 dBc/Hz, which is as much as 13 dB lower than for the laser we used in our current experiments), the noise figure of the HCF link is lower than when using SSMF. In the 10–15 GHz range, it is lower by 6 dB. For higher frequencies, the advantage becomes even higher (up to almost 8 dB), with the extra ~2 dB due to the higher HCF APL gain as compared to the SMF APL gain that suffers from the above-mentioned chromatic-dispersion-induced signal fading. The 6-dB advantage is due to the more advantageous position of the EDFA in the HCF link (in front of the fibre); this is not possible in SSMF due to SBS, requiring the EDFA to be placed after the fibre link. When the EDFA is in front of the transmission fibre (HCF APL), the generated ASE gets attenuated in the link, while for the EDFA after the link (SSMF APL), it enters into the PD, increasing the noise level.

### Dynamic range

**Fig. 8:**
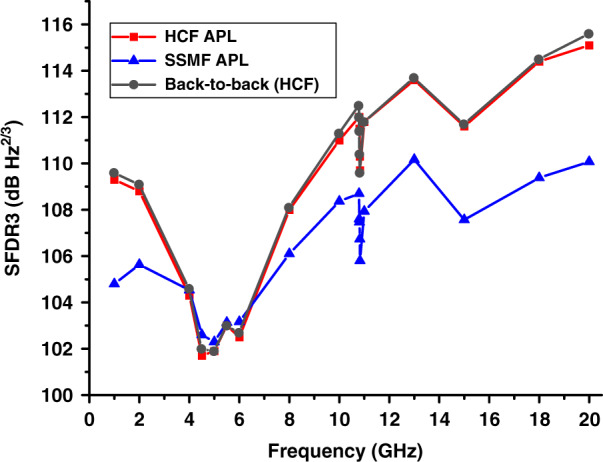
The measured SFDR3 of the noise figure optimized HCF (squares) and SSMF (triangles) APLs. For the HCF APL, the SFDR3 with the HCF replaced by an attenuator (back-to-back) is also shown (circles), confirming there is no degradation in the APL performance due to HCF impairments

As mentioned earlier, HCFs are not truly single-moded. Although higher-order modes can be, as we already mentioned, very lossy and thus in practice do not propagate over longer lengths, they can produce multi-path interference via distributed coupling between them and the fundamental mode, potentially increasing the noise level via time-varying multi-path interference. This would reduce the APL dynamic range. To investigate this, we measured the third-order spurious-free dynamic range (SFDR3) in the noise figure optimized HCF (gain and noise figure shown in Fig. 7), and compared it to the back-to-back measurement, in which the HCF was replaced with an optical attenuator with the same loss (Fig. 8). For comparison, we also show in Fig. 8 the SFDR3 of the noise figure optimized SSMF APL (gain and noise figure shown in Fig. 7). For SFDR3 measurement, we measured the third-order intermodulation power using the two-tone test method^44,45^, which uses a pair of closely spaced signal frequencies. Details of our implementation are included in the ‘Methods’ section. SFDR3 is then obtained from the measured fundamental power, third-order intermodulation power, and noise floor.

The measured SFDR3 shows a degradation around 5 GHz as the signal laser RIN has its peak there due to its relaxation oscillation that occurs at this frequency. The degradation around 10.8 GHz is mainly caused by SBS in the EDFAs. The SFDR3 is better than 101 dB·Hz^2/3^ over the entire range and achieves 115 dB·Hz^2/3^ at the frequency of 20 GHz for the HCF link. Crucially, we see SFDR3 to be essentially identical for the link with and without HCF, confirming that HCF does not bring any degradation in terms of distortion (SFDR3), even in an APL with an SFDR3 as high as 115 dB·Hz^2/3^.

### Potential for WDM APL

Thanks to the HCF’s low optical nonlinearity, several orders of magnitude lower than in SSMFs, HCF APLs should not suffer from FWM and thus be able to also support WDM APL. WDM APL would have several advantages over a single-channel system, e.g., increased capacity or ability to perform low-loss signal splitting, in which each wavelength is routed, e.g., to a different RHH.

**Fig. 9:**
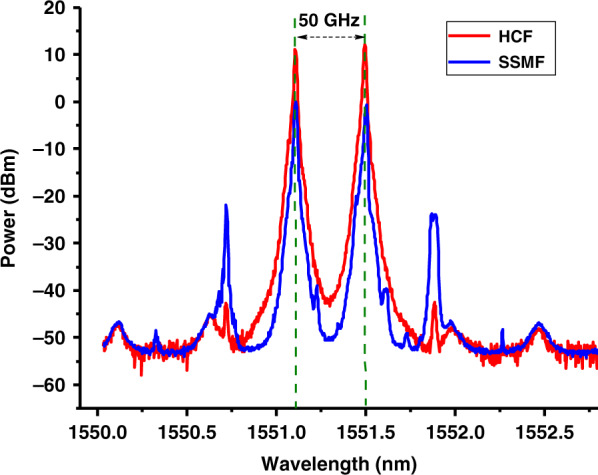
Measured FWM inside 7.7 km of HCF (red) and 7.4 km SSMF (blue). Two CW laser signals with wavelengths 0.4 nm (50 GHz) apart (their absolute wavelengths are marked with green dashed lines), each with 27 dBm power, are launched into the respective fibres. The output shows FWM products as new signals 50 GHz apart from the two input signals, They are about 20 and 55 dB weaker than the original signals in the SSMF and HCF cases, respectively, showing significantly weaker FWM interaction in the HCF as compared to the SSMF

To demonstrate this property, we launched two CW signals, 27 dBm each and spaced 50 GHz apart into the SSMF and HCF samples and observed their spectra at the fibre outputs (Fig. 9). While we observe appreciable FWM products in the SSMF (−20 dB below the signals), the small FWM observed at the HCF output (−55 dB below the signals) is due to FWM in the EDFA, which we have confirmed by measuring the spectrum at the EDFA output.

## Conclusions

We have proposed and demonstrated the use of HCFs for APLs. We have shown how the HCF’s unique properties, such as a high SBS threshold and low Kerr-induced nonlinearities, achieved simultaneously with low chromatic dispersion, improve the APL in all key metrics including the link loss, noise figure, spurious-free dynamic range, and potential for WDM transmission. Specifically, we show a link loss improvement of over 15 dB in a “traditional” quadrature-biased 7.7-km-long HCF APL as compared to a 7.5-km SMF APL. Simultaneously, the noise figure was improved by 6 dB. For a low-biased HCF APL, we show a net gain over the entire measurement range of 0–20 GHz, which would be very challenging to achieve in a SSMF APL link of the same length.

HCF technology is maturing very quickly, with a recently-reported attenuation of 0.22 dB/km interconnection to SSMF with a loss as low as 0.15 dB, and increasing commercial availability of field-deployable cables. At the same time, the power of transmitters and receivers is increasing, ensuring the performance of future HCF-based APLs will be unrivalled and it should be expected to meet the requirements of virtually all currently-envisaged applications. It could prove revolutionary, for example, in massive scaling of low-cost and low-power-consumption remote radio heads for emerging (beyond 5G) ultrahigh-speed wireless Internet access (even in densely-populated areas), simultaneously providing low and stable latency to support new and emerging applications such as the navigation of self-driven cars, and remote control of robots. Another example is the prospect of enabling a significant increase in the range of radio-telescope arrays that are looking, for example, for habitable exoplanets.

## Methods

### HCF sample

**Fig. 10:**
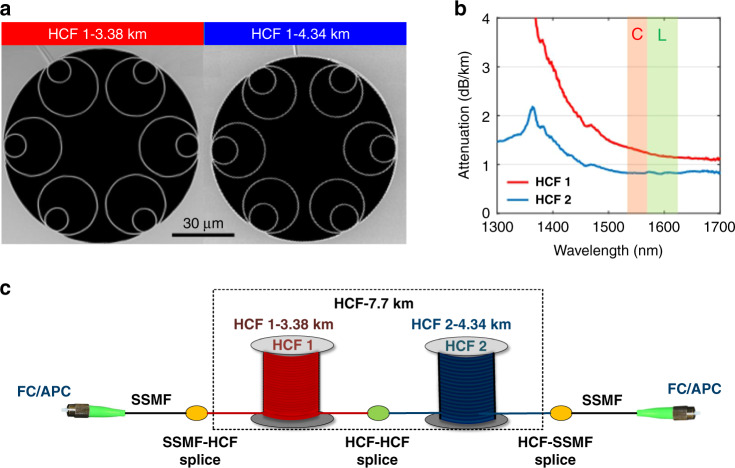
Fibre sample details. **a** Cross-sectional SEMs of the two HCF (NANF-type) samples. The length of the HCFs is 3.38 km and 4.34 km, respectively. **b** HCF attenuation versus transmission wavelength measured on both HCF samples with a cutback method. **c** Details of the 7.7-km HCF link. The loss of SSMF-HCF and HCF-HCF splices are 1 and 0.2 dB, respectively. FC/APC: fibre connector with angled physical contact

Our 7.72-km HCF link comprised two fibre spans, as shown in Fig. 10, both of NANF geometry and operating in the first antiresonant window^46^. The cross-sectional structure of the fibre in both spans was captured with the scanning electron microscope (SEM) and is shown in Fig. 10a. The two fibres are geometrically similar, with a difference in the size of their inner tubes. HCF 1 (3.38 km), has an average core diameter and membrane thickness of 35.6 and 0.55 μm, respectively. Its loss measured with a cutback method (Fig. 10b) decreases from 1.37 dB/km at 1530 nm to 1.15 dB/km at 1625 nm (1.30 dB/km at 1550 nm). As for HCF 2 (4.34 km), the corresponding parameters were 35.9 μm, 0.48 μm, and 0.82 dB/km, with the loss spectrally flat from 1530 to 1625 nm.

The fibres were spooled on bobbins with a diameter of 0.3 m. The attenuation of the assembled HCF line shown in Fig. 10c was 8.8 dB, which included the two HCF spans, mid-span HCF1-HCF2 splice (~0.2 dB), and two SSMF-HCF splices (~1 dB each) and that enabled HCF interconnection with standard fibre components. The total loss was 8.8 dB.

### SSMF sample

**Fig. 11:**
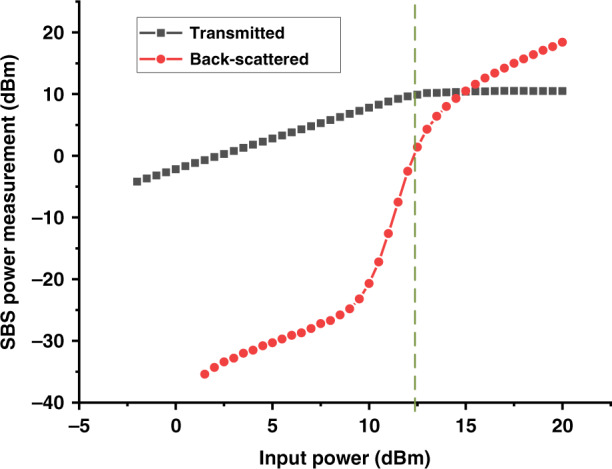
SBS threshold measurement result for 7.4 km SSMF Transmitted (squares) and back-scattered (circles) optical power versus launched optical power into the SSMF. The green dashed line indicates the measured SBS threshold of the 7.4 km SSMF

The transmitted and SBS-reflected power of our SSMF sample (7.4 km) as a function of the launched power is shown in Fig. 11. There are various definitions of SBS threshold in the literature^16,17,47–49^. Here we use the definition from ref. ^47^, where the SBS threshold is given by the maximum change in the slope (second derivative) of the reflected power (Fig. 11). For our SSMF sample, the SBS threshold was 12.5 dBm (Fig. 11).

### Experiment setups

#### Q-biased APL set-up displayed in Fig. 2

20 dBm of CW light power at 1550 nm from a DFB laser diode (with sub-MHz linewidth, relaxation oscillation around 5 GHz and with maximum RIN below −150 dBc/Hz) was modulated using a 20 GHz bandwidth MZM biased at its quadrature point. The power at the MZM output was 13 dBm. The signal was subsequently amplified to 27 dBm using an EDFA and attenuated to the required power level using a variable optical attenuator (VOA). Subsequently, the signal was launched into the 7.7 km HCF or 7.4 km SSMF fibres. At their output, the light was launched into a 20-GHz bandwidth photodiode with a maximum input power of 18 dBm. The received signal was then analysed with an electrical spectrum analyzer (ESA).

#### NF-optimized HCF APL noise figure measurement experiment shown in Fig. 5

20 dBm of CW light power at 1550 nm from a DFB laser diode was amplified to 27 dBm using an EDFA. Then the amplified signal was modulated using a 20 GHz bandwidth MZM biased at its low-biasing point. The output light with a power of 12 dBm was amplified by another EDFA to 33 dBm and launched into the 7.7 km HCF. The transmitted signal was split using a 20:80 optical coupler. The 80% output was attenuated to 18 dBm using a VOA and then detected with the 20-GHz bandwidth photodiode and analysed by an ESA. The 20% output was used for optical power monitoring using an optical power metre.

#### SBS-free SSMF APL experimental set-up shown in Fig. 6

20 dBm of CW light power at 1550 nm from a DFB laser diode was modulated using a 20 GHz bandwidth MZM biased at its low-biasing point. The power at the MZM output was 7.6 dBm. The signal was subsequently launched into the 7.4 km SSMF. The output signal was amplified using an EDFA to 27 dBm, attenuated to 18 dBm using a VOA, and finally detected with the 20-GHz bandwidth photodiode and analysed with the ESA.

### Noise level and noise figure measurement

**Fig. 12:**
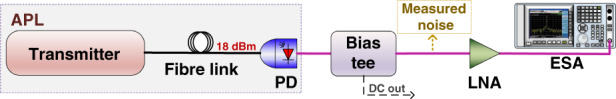
Set-up used to measure the noise level. PD, photodiode; ESA, electrical spectrum analyzer. The APL consists of a transmitter, fibre link and a PD. For noise-optimized APLs, the input optical power to the PD is set to its maximum value of 18 dBm. For noise level measurement, there is no RF input power to the transmitter, causing the received signal to be at DC, which is subsequently filtered out by the bias tee, leaving only the (AC) noise. This noise is then amplified by an LNA to amplify it above the ESA noise floor

The noise level was too weak to be measured directly by the ESA. Thus, we adopted the gain method^42^ in the set-up shown in Fig. 12. The optical power at the PD input was 18 dBm. However, there was no RF signal input at the MZM, such that the signal at the PD output was DC. We subsequently filter it out with a bias tee, leaving only the (AC) noise *p*_N_ at the input of a low-noise amplifier (LNA), HMC460 from Analog Devices, providing gain *g*_LNA_. This LNA amplified *p*_N_ above the noise floor of the ESA, *p*_ESA_. Consequently, the noise power measured by the ESA consisted of *p*_ESA_, amplified link noise *g*_LNA_*p*_*N*_, and noise from the LNA itself, *p*_LNA_. The noise from the APL link *p*_N_ is then calculated as

1 $$p_{\rm{N}} = \frac{{p_{{\rm{ESA}}} - \left( {p_{{\rm{SA}}} + p_{{\rm{LNA}}}} \right)}}{{g_{{\rm{LNA}}}}}$$

*p*_LNA_ is then calculated from the noise factor, *F*_LNA_ as

2 $$p_{{\rm{LNA}}} = g_{{\rm{LNA}}}\kappa T(F_{{\rm{LNA}}} - {{{\mathrm{1}}}})$$

where *κ* is the Boltzmann constant and *T* = 290 K is the room temperature. The noise factor is related to the LNA noise figure via the relation NF_LNA_ = 10 log *F*_LNA_.

The *p*_ESA_ power spectral density (PSD) was measured using the noise marker in the ESA set with a resolution bandwidth of 3 MHz. The marker was positioned at the desired offset RF frequency and the noise level was measured in a 30 kHz noise bandwidth and then re-normalized to 1 Hz bandwidth.

**Fig. 13:**
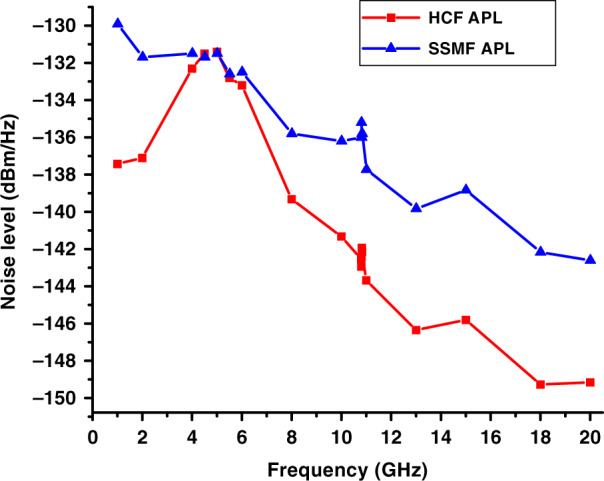
The measured noise level of noise figure optimized SSMF (triangle) and HCF (square) APLs over the frequency range of 0–20 GHz

The APL noise levels obtained from Eq. (1) for noise figure optimized SSMF and HCF APLs are shown in Fig. 13.

Then the link noise figure NF was calculated as:

3 $${\rm{NF}} = P_{\rm{N}}\left( {{\rm{dBm/Hz}}} \right) - G_{{\rm{link}}} + P_{{\rm{thermal}}}$$

where *P*_N_ = 10log10(*p*_N_), *G*_link_ is the link gain, and *P*_thermal_ is the thermal noise level of −174 dBm/Hz.

### SFDR3 measurement

**Fig. 14:**
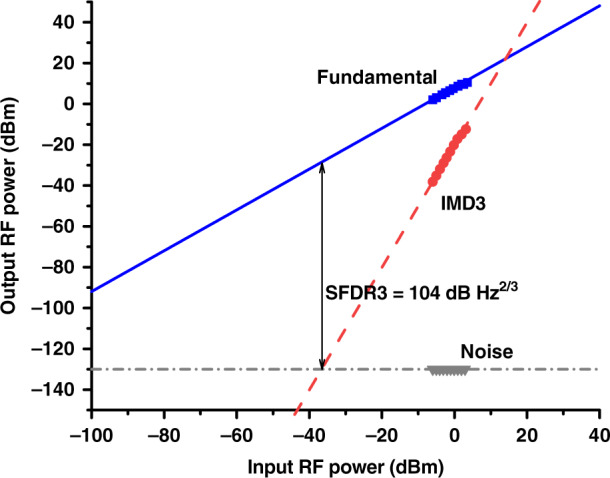
Example of the SFDR3 measurement

A two-tone RF signal with frequencies of *f*_1_ and *f*_2_ was generated by combining signals from two signal generators. The powers of the fundamental signal at *f*_1_ and *f*_2_ and the third-order intermodulation distortion (IMD3) at 2*f*_2_ − *f*_1_ were measured at the photodiode output using an ESA. The powers of the input RF signals were varied from −6 to 3 dBm with a step of 1 dBm and then the fundamental and IMD3 was measured. Subsequently, the IMD3 power, fundamental signal power, and noise level (obtained through Eq. (1) and as described in the previous section) were plotted, an example is shown in Fig. 14. SFDR3 is then determined as shown in Fig. 14^44^.

### Chromatic dispersion

**Fig. 15:**
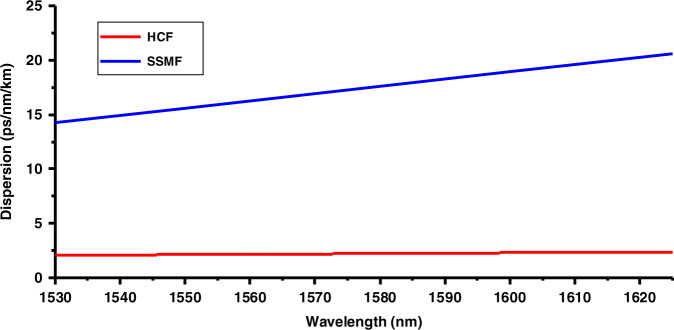
Measured chromatic dispersion results for 7.7 km HCF (red) and 7.4 km (blue) SSMF

We measured the chromatic dispersion of our HCF and SSMF using the phase shift method^50^. The results are shown in Fig. 15. As expected, the SSMF has a chromatic dispersion of 17 ps/nm/km at 1550 nm, while the HCF’s chromatic dispersion is only 2.1 ps/nm/km.
